# Replantation of Teeth

**Published:** 1882-04

**Authors:** G. J. Williams

**Affiliations:** Dental Surgeon to the Hospital.


### ARTICLE VI.

### *Replantation of Teeth.*

BY G. J. WILLIAMS, L. D. S., ENG., DENTAL SURGEON TO THE HOSPITAL.

[A Clinical Lecture delivered at the National Dental Hospital.]

*Gentlemen.*—I wish to bring before your notice this morning a patient whom we operated upon twelve months since, that will show you a successful replantation of a tooth; and, I may remark, it is seldom we have an authentic case that we can see so long after treatment. The replantation of teeth has been much discussed of late years, and opinions upon the procedure are divided. Many years have passed since Hunter proved that transplantation was possible by placing a tooth, recently extracted, in the comb of a cock; and, feeding the animal upon madder for some time, then killing the bird and finding the tooth coloured the same as the bones of the animal. It is recorded that some twenty years ago poor persons having good front teeth were prevailed upon to part with them for a monetary consideration, that they might be transplanted into other mouths, the teeth of which were faulty; but we have no reliable record of the success of the operation of late years. Where replantation of teeth has been done, it has been with a view of more successfully filling a cavity than could well be done whilst the tooth was in the mouth. The operation consists in the tooth having been carefully extracted and encircled in a damp piece of lint, the caries carefully removed, the cavities stopped, and the tooth, having been well washed in warm water, replaced in its socket and carefully tied to the two adjacent teeth. Some practitioners state that they are successful; whilst others are not so sanguine. As regards my own observations, I cannot say that what I have seen could be considered as successful; and I think longer time must ex pire between the operations and declaring the result than has usually been given, before success can safely be pronounced. For whilst the tooth may appear firm and satisfactory after a month or so, yet after six months or more the opinions would differ as to its success. During the early part of the time after t e operation the patient has necessarily been careful not to use the tooth much, as pain ensues if bitten upon a great deal, although the tooth attains a certain amount of firmness and ceases to give pain. It is frequently not until some time after replantation that, if carefully noticed, a difference in colour between the replanted tooth and its fellows will be observed. A darker or blacker hue will be noticed, due to a want of nourishment and consequent death of the tooth. Where this takes place we can hardly call the operation successful, even if twelve months have elapsed since the operation; and few teeth which have been called successfully treated have been that length of time under notice.

I have seen a few teeth that have been so treated, which, where any length of time has expired, have been more or less discoloured, a sure sign, I think, of ultimate failure. Of the operation itself, as regards the extraction of a decayed tooth with a view to stopping and replacing it in its socket, I candidly say I am not in favor of it. On the reasons I have given it is scarcely desirable, as being very doubtful in its success; but from other views it is not an operation I should advise, and if advised, I think I should find very few patients who would follow that advice. When a patient comes to you for treatment, it is generally with a view to save the torture of extraction, and to postpone that heroic treatment by stopping the tooth; and, if replanted, no positive assurance can be given that after much pain and inconvenience, a second extraction will not be required. As far as my experience goes, if a patient has once got rid of a troublesome member, he is in no hurry to have a repetition of the same description of suffering; and if the tooth has not been troublesome, but simply decayed, his visit to you is to get the tooth plugged, so as to prevent it becoming troublesome; but, in such case, to advise extraction as a primary move is, as I before remarked, heroic. I do not say no occasion may arise when it would be unwise not to do so, but, as a general rule, I think you will do best to perfect yourselves in filling the teeth whilst *in situ*, no matter how difficult the position of the cavity may be, for therein will lie your skill as pluggers of teeth.

There are occasions in which the replanting of teeth is not only desirable but imperative, and where success is most likely to ensue. It will sometimes occur where the first lower molar is decayed on its anterior third, that the remaining portion gets pushed forward by the molar teeth behind until the posterior part of the second bicuspid lies in the carious cavity of the molar, and the lower border of enamel will impinge immediately beneath the enamel of the bicuspid. To remove only the molar, unless the fangs are divided, or easily done so, is almost an impossibility; and upon the attempt to do so being made, the bicuspid, which is the easiest tooth to extract owing to the very tapering form of the fang, will spring out of its socket some little time before you get the molar away; and, when this accident does occur, by the immediate replacement of the bicuspid in its socket, if healthy, it will generally become a firm and useful member again, and keep its colour as though it had never been removed. If by any possibility such event occurs to any of you, at once replace the tooth, and entertain hope of good results.

The patient before us this morning has been treated somewhat in this way, and from the length of time that has passed since the operation was performed, I have thought it a good case to bring before your notice. On December 29th, 1880, the patient, a rather weakly, nervous girl, about 18 years of age, presented herself here for treatment. Her face was very much swollen, and great nervous prostration was visible. The history she gave was, that she had been to several places, the last being St. Bartholomew's Hospital, to have the stump of a tooth extracted. On each occasion they all punished her very much, but had failed to give her relief or extract the offender. Upon examination of her mouth, as some of you may remember, great difficulty was experienced in opening it wide enough, owing to the swelling and pain it caused. A very crowded state of teeth was seen, the absence of the crown of the second lower bicuspid was noticed, the fang being found deep in the alveolus. The six-year-old molar had been forced over the fang and was very near the first lower bicuspid. Now it was evident that, until the fang of the second bicuspid was removed, that being the one which was causing the disturbance (the other teeth being sound,) no relief could be found; and it was also evident that the fang could not be removed through the small opening above it, and it was the several attempts to do this that had so punished the patient to no avail. I deemed it desirable to first extract the first bicuspid, then remove the stump of the second bicuspid, replacing the first bicuspid in its place immediately; the whole operation, occupying some forty seconds. Directions were given to foment well the parts with warm poppy lotions, and the patient was sent away. If you examine the tooth now, being over twelve months since its replacement, you will find it firm, the same colour as its fellows, and likely to be amongst the last to be removed from its refound socket.—*London Dent. Record.*

